# FORECASTING AND COPING WITH FUTURE STRESSORS PREDICTS DAILY SUBJECTIVE AGE

**DOI:** 10.1093/geroni/igz038.202

**Published:** 2019-11-08

**Authors:** Shevaun D Neupert, Shevaun D Neupert, Xianghe Zhu

**Affiliations:** North Carolina State University, Raleigh, North Carolina, United States

## Abstract

Stressors are associated with higher subjective ages, but the role of forecasting and coping with future stressors is unknown. 223 adults (107 aged 18-36, 116 aged 60-90) reported their subjective age, forecasts of next-day health stressors, and anticipatory coping with next-day health stressors each day for eight consecutive days. There was no main effect of forecasting, but increases in plan rehearsal coping were associated with increases in felt age. In contrast, increases in problem analysis coping were associated with decreases in felt age. Daily forecasting and coping also interacted with each other. On days with low plan rehearsal or low problem analysis, there was no association between forecasting of health stressors and subjective age. However, on days with high plan rehearsal or high problem analysis, increases in forecasting ratings were associated with increases in subjective age. Forecasting and coping with future stressors may play a role in subjective aging.

